# Serological follow-up of SARS-CoV-2 asymptomatic subjects

**DOI:** 10.1038/s41598-020-77125-8

**Published:** 2020-11-18

**Authors:** Gregorio Paolo Milani, Laura Dioni, Chiara Favero, Laura Cantone, Chiara Macchi, Serena Delbue, Matteo Bonzini, Emanuele Montomoli, Valentina Bollati, the UNICORN Consortium, Benedetta Albetti, Claudio Bandi, Tommaso Bellini, Marco Buscaglia, Carlo Cantarella, Michele Carugno, Sergio Casartelli, Sarah D’Alessandro, Francesca De Chiara, Ivano Eberini, Luca Ferrari, Monica Ferraroni, Laura Galastri, Cristina Galli, Mirjam Hoxha, Simona Iodice, Carlo La Vecchia, Alessandro Manenti, Ilaria Manini, Serena Marchi, Jacopo Mariani, Elena Pariani, Angela Cecilia Pesatori, Federica Rota, Massimiliano Ruscica, Tommaso Schioppo, Letizia Tarantini, Claudia Maria Trombetta, Marco Vicenzi, Giuliano Zanchetta

**Affiliations:** 1 Department of Clinical Sciences and Community Health, Università degli Studi di Milano, Milan, Italy; 2 Pediatric Unit, Fondazione IRCCS Ca’ Granda Ospedale Maggiore Policlinico, Milan, Italy; 3 EPIGET Lab, Department of Clinical Sciences and Community Health, Università degli Studi di Milano, Milan, Italy; 4 Department of Pharmacological and Biomolecular Sciences (DiSFeB), Università degli Studi di Milano, Milan, Italy; 5 Department of Biomedical, Surgical and Dental Sciences, Laboratory of Translational Research, Via Carlo Pascal 36, 20133 Milano, Italy; 6 Occupational Health Unit, Fondazione IRCCS Ca’ Granda Ospedale Maggiore Policlinico, 20122 Milan, Italy; 7 Department of Molecular and Developmental Medicine, University of Siena, Siena, Italy; 8 Department of Biosciences and Pediatric Clinical Research Center “Romeo and Enrica Invernizzi”, University of Milan, Milan, Italy; 9 Department of Medical Biotechnology and Translational Medicine, University of Milan, 20129 Milan, Italy; 10 AVIS (Associazione Volontari Italiani Sangue) Milano, Milan, Italy; 11 Branch of Medical Statistics, Biometry, and Epidemiology “G. A. Maccacaro”, Department of Clinical Sciences and Community Health, Università degli Studi di Milano, Milan, Italy; 12 Department of Biomedical Sciences for Health, University of Milan, Milan, Italy; 13 VisMederi Research Srl, Siena, Italy; 14 Division of Rheumatology, ASST Pini-CTO, Milan, Italy; 15 Department of Internal Medicine, Fondazione IRCCS Ca’ Granda Ospedale Maggiore Policlinico, 20122 Milan, Italy; 16 Dyspnea Lab, Department of Clinical Sciences and Community Health, University of Milan, Milan, Italy

**Keywords:** Biomarkers, Epidemiology

## Abstract

SARS-CoV-2 symptoms are non-specific and can range from asymptomatic presentation to severe pneumonia. Asymptomatic subjects carrying SARS-CoV-2 often remain undiagnosed and it is still debated whether they develop immunoglobulins (Ig) and how long they persist. The aim of this study was to investigate the development and persistence of antibodies against SARS-CoV-2 in asymptomatic subjects infected by the virus. This follow-up study was performed on the 31 asymptomatic subjects who presented a positive nasal swab or serology against SARS-CoV-2 (Ig against Spike-RBD) in the first part of the UNICORN study (March 2020) aimed at attesting previous or current contacts with the virus in the personnel of the University of Milan. Eight weeks after the first Ig measure, these subjects were invited to donate a second blood sample for testing serum antibodies (IgM, IgG and total antibodies) and to fill-in a structured questionnaire. About 80% of asymptomatic subjects did not present circulating immunoglobulins against SARS-CoV-2 after 8 weeks from a positive nasal swab against the virus. Moreover, in more than 40% of these subjects, no Ig against SARS-CoV-2 were detected at any time. Finally, about two third of subjects with immunoglobulins at baseline did not present IgG against SARS-CoV-2 after 8 weeks. The majority of subjects who developed an asymptomatic SARS-CoV-2 infection do not present antibodies against the RBD-spike protein after 8 weeks of follow-up. These data should be taken into account for the interpretation of the serological evidences on SARS-CoV-2 that are emerging nowadays.

## Introduction

Coronaviruses are known to cause diseases ranging from the common cold to fatal infections^1^. Among these viruses, the SARS-CoV-2 is responsible for the current infectious outbreak that has been declared a pandemic public health emergency by the World Health Organization.

SARS-CoV-2 symptoms are non-specific and can range from no symptoms to severe pneumonia^2^. This makes of primary importance to profile individual characteristics, such as the variability in immune response, linked to the relevance of clinical signs. Asymptomatic subjects carrying SARS-CoV-2 often remain undiagnosed and it is still debated whether they are able to transmit the disease^3,4^ and develop immunoglobulins (Ig)^5^.

Ig reveal evidence of a previous infection from about a week after the infection occurred^6^, but to date it is not clear if they are produced by all subjects encountering the virus and how long they persist in blood. Moreover, the actual capacity of anti-SARS-CoV-2 Ig to be neutralizing antibodies is still under debate^7–9^, especially for asymptomatic subjects. Many different methods have been proposed to detect Ig against SARS-CoV-2. To date, the most promising antigen for serodiagnosis of COVID-19 is probably the spike (S) protein, in particular the receptor-binding domain (RBD) mediating the interaction with angiotensin-converting enzyme 2 (ACE2)^10,11^.

At the end of March 2020, we examined plasma samples from 197 asymptomatic (at recruitment and in the 14 days before) subjects, enrolled during the lockdown period in Milan (Italy)^12^. This study was the first part of the UNICORN (“UNIversity against CORoNavirus”) project that was conducted among the personnel of the University of Milan, the largest university in Lombardy (Italy). A total of 31 subjects (16%) presented at least one positive test attesting a previous or current contact with SARS-CoV-2. In particular, 10% presented antibodies (IgM or IgG) against SARS-CoV-2 and the SARS-CoV-2 RNA was detected in the nasal swab of 21 subjects (11%)^12^.

The aim of the study was to investigate the development or persistence of antibodies against the spike-RBD among the 31 asymptomatic subjects, 8 weeks after the first sampling.

## Methods

In this follow-up study, the 31 subjects who presented a positive nasal swab or serology against SARS-CoV-2 in the first part of the UNICORN project (T1)^12^ were eligible.

Eight weeks after the first sampling (T2), these individuals were invited to donate a second blood sample and to fill-in a structured questionnaire.

The study was approved by the ethics committee of the University of Milan (approval number 17/20, approval date March 6, 2020) and conducted in accordance with the Declaration of Helsinki. All participants signed an informed consent form.

### Blood collection and Ig analyses

Blood was collected in ethylenediaminetetraacetic acid (EDTA) tubes (9.5 ml), and transported to the EPIGET Lab (University of Milan) within 2 h after phlebotomy. Blood-EDTA was processed to separate buffy coat and plasma, by centrifuging at 1200×*g* for 15 min at room temperature. Cell-free plasma was used to assess immunoglobulin-M (IgM) and immunoglobulin-G (IgG) against SARS-CoV-2 using validated enzyme linked immunosorbent assay (ELISA) methods.

The Wantai anti-SARS-CoV-2 IgM ELISA (Beijing Wantai Biological Pharmacy Enterprise, Beijing, China)^13^ were performed according to the manufacturer’s instructions. Reported sensitivity is 0.86 and specificity is 1. The assays detect antibodies binding SARS-CoV-2 spike protein receptor binding domain (RBD) in human serum or plasma. Briefly, 10 μl plasma samples and 100 μl of Specimen Diluent were added to wells coated with antibodies directed against the human immunoglobulin M proteins, and incubated for 30 min at 37 °C. Each well was aspirated and washed five times using an automatic microplate washer (MicroFill Dispenser, BioTek Instrument Winooski, VT, USA). Then, a recombinant HRP-conjugated SARS-CoV-2 antigen was added and incubated for 30 min at 37 °C. After a further washing, a chromogen solution was added. The reaction was stopped after 15 min at 37 °C, and the resultant absorbance was read on a microplate reader (Synergy HT, BioTek Instrument) at 450 nm with reference at 620 nm. The cut-off value for a positive result was calculated according to the manufacturer’s instruction, and equal to 0.105 for Anti-SARS-CoV-2 IgM ELISA.

To perform RBD Enzyme-Linked Immunosorbent Assay (ELISA) IgG, ELISA plates were coated with 1 µg/mL of purified recombinant Spike-RBD HEK-derived protein (Sino Biological, China). After overnight incubation at + 4 °C, coated plates were washed three times with 300 µl/well of ELISA washing solution containing Tris Buffer Saline (TBS)-0.05% Tween 20, then blocked for 1 h at 37 °C with a solution of TBS containing 5% of Non-Fat Dry Milk (NFDM) ( Euroclone, Pero, Italy)^14^.

Human serum samples were heat inactivated at 56 °C for 1 h in order to reduce the risk of the presence of live virus in the sample, then diluted 1:100 in TBS-0,05% Tween 20 5%. Plates were washed three times as previously than 100 µl of each serum dilution was added to the coated plates and incubated for 1 h at 37 °C. Next, after the washing step, 100 µl/well of Goat anti-Human IgG-Fc HRP-conjugated antibody diluted 1:100,000, (Bethyl Laboratories, Montgomery USA) were added. Plates were incubated at 37 °C for 30 min. Following incubation, the plates were washed and 100 µl/well of 3,3′,5,5′-Tetramethylbenzidine (TMB) substrate (Bethyl Laboratories, Montgomery, USA) was added and incubated in the dark at room temperature for 20 min. The reaction was stopped by adding 100 µl of ELISA stop solution (Bethyl Laboratories, Montgomery, USA) and read at 450 nm. Cut-off value was established as 3 times the average of optical density (OD) values from blank wells (background-no addition of analyte). Samples with the ODs under the cut off value at the first (1:100) dilution were assigned as negative, samples where the ODs at 1:100 dilution were above the cut-off value were assigned as positive. Borderline samples were defined where one replicate was under the cut-off and the other was above^14^.

### Statistical analyses

We used standard descriptive statistics to summarize data. Categorical data were presented as frequencies and percentages. Continuous variables were expressed as the mean ± standard deviation (SD) or as the median and interquartile range [Q1–Q3], as appropriate. To investigate the characteristics of study participants who had Ig G against Sars-CoV-2 at T2, the Fisher exact test for categorical variables and t-test or Wilcoxon sum-rank test for continuous variables, were used. We reported the odds ratio and 95% confidence intervals (CI) to evaluate the association between SARS-Cov-2 RNA, IgM and IgG measured at T1 with IgG measured at T2. Statistical analyses and graph were performed with SAS software (version 9.4; SAS Institute Inc., Cary, North Carolina, USA, https://www.sas.com) and R software (version 3.6.1; Foundation for Statistical Computing, Vienna, Austria, https://www.r-project.org/).

### Ethics committee approval

The study was approved by the ethics committee of the University of Milan (approval number 17/20, approval date March 6, 2020) and conducted in accordance with the Declaration of Helsinki.

## Results

Among the 31 eligible subjects, 29 (94%) completed the follow-up study, while 2 subjects revoked their participation before T2. Characteristics of the 29 volunteers (17 females and 12 males, median age 44 years) are described in Table 1. Most subjects had high-level education: all participants had at least a higher school degree (high school: 20.7%; university: 20.7%, postgraduate: 58.6%). 79% had at least one cohabiting family member, and in 34.5% of cases the family member was < 10-year-old.

**Table 1:** Characteristics of the study participants.

	All subjects	Subjects with positive T2 IgG	Subjects with negative T2 IgG	P-value
	N = 29	N = 7	N = 22	
**Age, years median [Q1-Q3]**	44 [37–59]	40 [37–53]	47.5 [37–60]	0.4143 ◊
**Gender, N (%)**				
Males	12 (41.4)	2 (28.6)	10 (45.45)	0.6645*
**BMI, kg/m**^**2**^** mean ± SD**	22.94 ± 2.49	22.43 ± 1.83	23.12 ± 2.7	0.5347○
**Smoking, N (%)**				
Never	13 (44.8)	5 (71.4)	8 (36.4)	0.4509*
Former	6 (20.7)	1 (14.3)	5 (22.7)	
Current	8 (27.6)	1 (14.3)	7 (31.8)	
Missing	2 (6.9)	-	2 (9.1)	
**Education, N (%)**				
High school	6 (20.7)	0 (0.0)	6 (27.3)	0.1661*
University	6 (20.7)	3 (42.9)	3 (13.6)	
Above University	17 (58.6)	4 (57.1)	13 (59.1)	
**Cohabiting with at least one family member, N (%)**	23 (79.3)	5 (71.4)	18 (81.8)	0.4813*
**Cohabiting family members, median [Q1-Q3]**	2^1,3^	3 [0—4]	2^1,2^	0.5033 ◊
**At least one child younger than 10 years old, N (%)**	10 (34.5)	3 (42.9)	7 (31.8)	0.6119*
**Residence area, N (%)**				
City	22 (75.9)	6 (85.7)	16 (72.6)	1*
Peripheral area	1 (3.5)	0 (0.0)	1(4.6)	
Village/small city	3 (10.3)	1 (14.3)	2 (9.1)	
Rural area	1 (3.5)	0 (0.0)	1(4.6)	
Missing	2 (6.9)	-	2 (9.1)	
**Lifestyle, N (%)**				
Sedentary	7 (24.1)	1 (14.3)	6 (27.3)	0.6107*
Active	12 (41.4)	4 (57.1)	8 (36.4)	
Sporty	3 (10.3)	0 (0.0)	3 (13.6)	
Active and sporty	5 (17.2)	2 (28.6)	3 (13.6)	
Missing	2 (6.9)	-	2 (9.1)	
**Flu vaccine, N (%)**				
Yes	8 (27.6)	2 (28.6)	6 (27.3)	0.9466*
**From October 2019**				
**Upper airway infections, N (%)**				
Yes	19 (65.5)	4 (57.4)	15 (68.2)	0.6647*
**Lower airway infections, N (%)**				
Yes	1 (3.5)	1 (14.3)	0 (0.00)	0.2414*
**Fever, N (%)**				
Yes	11 (37.9)	3 (42.9)	8 (36.4)	1*
**At least one of symptoms, N (%)**				
Yes	20 (69.0)	5 (71.4)	15 (68.2)	1*
**Chronic diseases,** N (%)				
Diabetes	2 (6.9)	0 (0.0)	2 (9.1)	1*
Hypertension	1 (3.5)	0 (0.0)	1(4.6)	1*
Chronic obstructive pulmonary disorder	1 (3.5)	0 (0.0)	1(4.6)	1*
Asthma	1 (3.5)	0 (0.0)	1(4.6)	1*
Cardiovascular disease	0 (0.0)	0 (0.0)	0 (0.0)	-
Chronic liver disease	1 (3.5)	0 (0.0)	1(4.6)	1*
Chronic neurological disease	0 (0.0)	0 (0.0)	0 (0.0)	-
Autoimmune disease	2 (6.9)	1 (14.3)	1(4.6)	0.4310*
Cancer	1 (3.5)	0 (0.0)	1(4.6)	1*
Others	2 (6.9)	0 (0.0)	2 (9.1)	1*
**Medications (continuative use in the last 6 months), N (%)**				
Antihypertensive	1 (3.5)	0 (0.0)	1(4.6)	1*
Corticosteroids	1 (3.5)	1 (14.3)	0 (0.0)	1*
Immunosuppressants	0 (0.0)	0 (0.0)	0 (0.0)	-
Chemotherapy	0 (0.0)	0 (0.0)	0 (0.0)	-

Continuous variables are expressed as mean ± standard deviation (SD) or as median [first quartile-third quartile] if not normally distributed; discrete variables are expressed as counts (%).

*BMI* Body Mass Index; *Q1* first quartile, *Q3* third quartile.

*P-value from Fisher exact test.

^◊^P-value from Wilcoxon rank-sum test.

^○^P-value from t-test.

**Figure 1:**
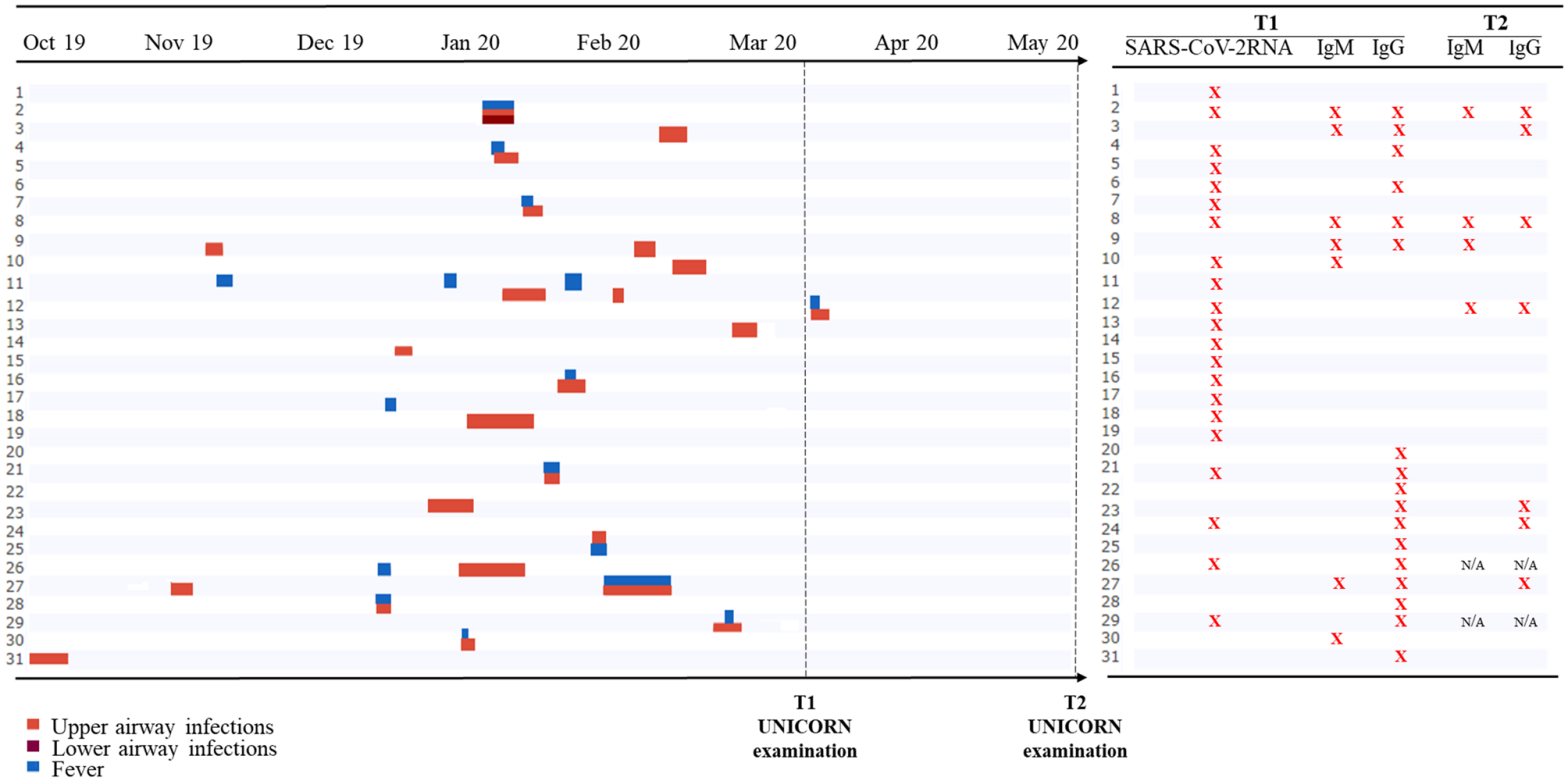
Left panel, a timeline showing symptoms occurrence from October 2019 to the second UNICORN examination (May-June 2020). Right panel, for each subject the presence/absence of Ig or viral RNA is reported.

In the 6 months preceding T1, 67.7% of the enrolled volunteers reported at least one episode of upper airway infections, 3.5% of lower airway infections, and 37.9% of fever (Table 1 and Fig. 1). However, none required hospitalization. One subject developed fever and symptoms consistent with an upper respiratory infection two day after the first nasal swab sampling.

Eleven (52.3%) out of 21 subjects with a positive nasal swab at T1 never developed antibodies against SARS-CoV-2. Antibody production was not associated to viral load (Supplementary Table 1).

Only 4 (21%) out of 19 participants (as 2 subjects were lost at T2) presented with immunoglobulins against SARS-CoV-2 at T2. Six (35%) out of 17 subjects with immunoglobulins at T1 presented IgG against SARS-CoV-2 at T2 (Fig. 1).

Subjects showing positive IgG at T2 did not differ from negative subjects for demographics and the number of upper and lower respiratory infections in the previous months (Table 1).

The presence of IgM at T1 or having 2 or more positive markers (among viral RNA, IgM or IgG) at T1 increase more than eightfold the probability of having positive IgG at T2 (respectively, OR 8.44, 95% CI (1.23 ; 58.16), p-value = 0.03; OR 8.50, 95% CI (1.25 ; 57.92), p-value = 0.03). On the contrary, the presence of positive IgG at T2 is not associated to the presence of viral RNA at T1 (OR 0.62, 95% CI (0.11 ; 3.56), p-value = 0.59). No other variables (listed in Table 1) are associated to the probability of having positive IgG at T2 (*data not shown*).

## Discussion

This study points out that about 80% of subjects with a mild SARS-CoV-2 infection do not present circulating immunoglobulins against SARS-CoV-2 after 8 weeks from a positive nasal swab against the virus. Moreover, in more than 40% of these subjects, no Ig against SARS-CoV-2 were detected. Finally, about two third of subjects with immunoglobulins at baseline did not present IgG against SARS-CoV-2 after 8 weeks.

Patients with severe forms of COVID-19 were demonstrated to develop immunoglobulins against the S protein within 2–3 weeks after the symptoms onset^15,16^. Very recent data also suggest that circulating immunoglobulins against the S protein in symptomatic patients tend to persist at least for three months^10^. Our data appear to show a discrepancy from what was previously observed in patients with symptomatic forms of SARS-CoV-2. Only a minority of subjects who were positive for viral RNA at T1 then developed IgG (38%), and only a part of them then maintained a measurable level of IgG after 8 weeks (14,3%). This apparent difference could be explained by the fact that the populations under study are different as regards the severity of the symptoms. We speculate that asymptomatic/paucisymptomatic positive subjects might benefit of some protective factors capable of limiting the spread of the virus within the body, or alternatively a more effective innate immune T cell-based response, as recently raised by Grifoni et al., 2020^17^. These factors could reduce the need for an adaptive response by the body, and consequently limit the production and persistence of antibodies.

Our data are in line with two very recent reports. In non-severe symptomatic COVID-19 patients, a decrease in IgG against RBD-spike protein was observed approximately 3 months after symptoms onset^18^. Moreover, a weaker immune response to SARS-CoV-2 infection was demonstrated in asymptomatic/paucisymptomatic individuals compared to symptomatic patients^19^.

To estimate the percentage of subjects who have been infected by SARS-CoV-2, an increasing number of studies have been conducted to assess the seroprevalence of antibodies against SARS-CoV-2 among the general population^20–23^. It has been proposed that these data may represent a starting point to predict how viral diffusion will evolve in the coming months^24^. At the light of our results, this assumption should be made with great caution. Considering the transient persistence of IgG in the asymptomatic/paucisymptomatic subjects, and that SARS-CoV-2 infection very often presents without symptoms, seroprevalence might not correspond to the real spread of the virus over time. Furthermore, to date there is no information available on the real protection from future infections in anti-SARS-CoV-2 IgG positive subjects.

Surprisingly, the only presence of SARS-CoV-2 RNA in the nasal swab at baseline was not associated with the probability of having anti- SARS-CoV-2 IgG after 8 weeks, with an odds ratio that was even protective, although not significant. On the contrary, the presence of IgM at T1 is associated with a strong increase in the probability of having IgG at T2. Although this association is significant, it is important to point out that the confidence intervals for the odds ratios are extremely wide and therefore this finding need to be interpreted cautiously. Moreover, since the analysis of the swab only detects the presence of viral RNA in the upper airways, this might not reflect a real active replication of the virus^25,26^, whereas Ig development implies an interaction between the virus and the immune system.

This study has some limitations. First of all, the population under study is small, although well characterized. The first part of the UNICORN project (the end of March, 2020) was conducted in Milan during a strict lockdown, and therefore subjects’ enrolment was limited. Second, the follow-up of positive subjects was relatively short. Finally, we did not perform any neutralizing test, although Ig against SARS-CoV-2 spike are the main, and likely the only neutralizing antibodies^27^.

In conclusion, this study points out that the majority of subjects who developed a mild SARS-CoV-2 infection do not present antibodies against the RBD-spike protein after 8 weeks of follow-up. These findings should be taken into account for the interpretation of seroprevalence data that are emerging nowadays.

## Supplementary information

Supplementary Table 1.
