# Supplementary material for: Serological follow-up of SARS-CoV-2 asymptomatic subjects
Source: Sci Rep. 2020 Nov 18;10:20048. doi: 10.1038/s41598-020-77125-8 (PMC7674414; doi:10.1038/s41598-020-77125-8)
Supplement: Supplementary file 1 — Supplementary Table 1. [file 41598_2020_77125_MOESM1_ESM.docx]

**Supplementary information**

**Serological follow-up of SARS-CoV-2 asymptomatic subjects**

Gregorio Paolo Milani^1,2^, Laura Dioni^3^, Chiara Favero^3^, Laura Cantone^3^, Chiara Macchi^4^, Serena Delbue^5^, Matteo Bonzini^3,6^, Emanuele Montomoli^7^, Valentina Bollati^3^, the UNICORN Consortium *

^1^ Department of Clinical Sciences and Community Health, Università degli Studi di Milano, Milan, Italy;

^2^ Pediatric Unit, Fondazione IRCCS Ca’ Granda Ospedale Maggiore Policlinico, Milan, Italy;

^3^ EPIGET Lab, Department of Clinical Sciences and Community Health, Università degli Studi di Milano, Milan, Italy.

^4^Department of Pharmacological and Biomolecular Sciences (DiSFeB), Università degli Studi di Milano, Milan, Italy.

^5^ Department of Biomedical, Surgical and Dental Sciences, Laboratory of Translational Research, Via Carlo Pascal 36, 20133 Milano, Italy.

^6^ Occupational Health Unit, Fondazione IRCCS Ca' Granda Ospedale Maggiore Policlinico, 20122 Milan, Italy.

^7^ Department of Molecular and Developmental Medicine, University of Siena, Siena, Italy.

*The full list of UNICORN consortium members included also the following investigators (in alphabetical order):

Benedetta Albetti^3^, Claudio Bandi^8^, Tommaso Bellini^9^, Marco Buscaglia^9^, Carlo Cantarella^3^, Michele Carugno^3,6^, Sergio Casartelli^10^, Sarah D’Alessandro^5^, Francesca De Chiara^10^, Ivano Eberini^4^, Luca Ferrari^3^, Monica Ferraroni^11^, Laura Galastri^10^, Cristina Galli^12^, Mirjam Hoxha^3^, Simona Iodice^3^, Carlo La Vecchia^11^, Alessandro Manenti^13^, Ilaria Manini^5^, Serena Marchi^5^, Jacopo Mariani^3^, Elena Pariani^12^, Angela Cecilia Pesatori^3,6^, Federica Rota^3^, Massimiliano Ruscica^4^, Tommaso Schioppo^14^, Letizia Tarantini^3^, Claudia Maria Trombetta^7^, Marco Vicenzi^15,16^, Giuliano Zanchetta^9^

UNICORN Consortium contact: [unicorn@unimi.it](mailto:unicorn@unimi.it)

^8^ Department of Biosciences and Pediatric Clinical Research Center "Romeo and Enrica Invernizzi", University of Milan, Milan, Italy.

^9^ Department of Medical Biotechnology and Translational Medicine, University of Milan, Milan, 20129, Italy.

^10^ AVIS (Associazione Volontari Italiani Sangue) Milano, Milan, Italy.

^11^ Branch of Medical Statistics, Biometry, and Epidemiology "G. A. Maccacaro", Department of Clinical Sciences and Community Health, Università degli Studi di Milano, Milan, Italy.

^12^ Department of Biomedical Sciences for Health, University of Milan, Milan, Italy.

^13^ VisMederi Research Srl, Siena, Italy.

^14^ Division of Rheumatology, ASST Pini-CTO, Milan, Italy.

^15^ Department of Internal Medicine, Fondazione IRCCS Ca' Granda Ospedale Maggiore Policlinico, 20122 Milan, Italy.

^16^ Dyspnea Lab, Department of Clinical Sciences and Community Health, University of Milan, Milan, Italy.

**Supplementary Table 1**: Viral load for SARS-CoV-2 positive subjects. Results were obtained as follows:
For each assay, Ct<37: positive; 37≤Ct≤40: repeat the test; Ct = Undetermined or = 40: negative.
If any two of the three assays are positive then SARS-CoV-2 RNA is present.
If any one of the assays is positive in two different samples (here named FIRST RUN and SECOND RUN), then SARS-CoV-2 RNA is present.
If all three of the assays are negative, then SARS-CoV-2 RNA is not present.

|  | **FIRST RUN** | | | | | | **SECOND RUN** | | | | | |
| --- | --- | --- | --- | --- | --- | --- | --- | --- | --- | --- | --- | --- |
|  | **Ct** | | | **n° copies/µL** | | | **Ct** | | | **n° copies/µL** | | |
| **Sample #** | **S** | **N** | **ORF1ab** | **S** | **N** | **ORF1ab** | **S** | **N** | **ORF1ab** | **S** | **N** | **ORF1ab** |
| **1** | Undetermined | 27.26 | 19.11 | ND | 37.99 | 502.15 | n/a | n/a | n/a | ND | ND | ND |
| **2** | 27.46 | 19.89 | 23.45 | 5.01 | >1000 | 51.37 | n/a | n/a | n/a | ND | ND | ND |
| **4** | Undetermined | 30.57 | Undetermined | ND | 5.05 | ND | Undetermined | 33.96 | Undetermined | ND | 0.38 | ND |
| **5** | Undetermined | 29.15 | Undetermined | ND | 12.06 | ND | Undetermined | 31.34 | Undetermined | ND | 2.17 | ND |
| **6** | Undetermined | 33.50 | Undetermined | ND | 0.84 | ND | Undetermined | 35.84 | Undetermined | ND | 0.11 | ND |
| **7** | Undetermined | 21.25 | Undetermined | ND | >1000 | ND | Undetermined | 29.35 | 30.58 | ND | 8.16 | 4.85 |
| **8** | Undetermined | 30.51 | Undetermined | ND | 5.25 | ND | Undetermined | 23.50 | Undetermined | ND | > 1000 | ND |
| **10** | Undetermined | 20.47 | Undetermined | ND | >1000 | ND | Undetermined | 29.78 | Undetermined | ND | 6.14 | ND |
| **11** | Undetermined | 27.97 | 17.52 | ND | 24.72 | >1000 | n/a | n/a | n/a | ND | ND | ND |
| **12** | 17.08 | 15.66 | 15.18 | >1000 | > 1000 | >1000 | n/a | n/a | n/a | ND | ND | ND |
| **13** | Undetermined | 29.63 | Undetermined | ND | 8.97 | ND | Undetermined | 31.89 | Undetermined | ND | 1.50 | ND |
| **14** | Undetermined | 28.44 | 19.91 | ND | 18.56 | 329.91 | n/a | n/a | n/a | ND | ND | ND |
| **15** | Undetermined | 25.53 | Undetermined | ND | 109.49 | ND | Undetermined | 30.58 | Undetermined | ND | 3.61 | ND |
| **16** | Undetermined | 26.85 | Undetermined | ND | 49.15 | ND | Undetermined | 37.48 | Undetermined | ND | 0.06 | ND |
| **17** | Undetermined | 20.10 | Undetermined | ND | >1000 | ND | Undetermined | 32.43 | Undetermined | ND | 1.05 | ND |
| **18** | Undetermined | 30.54 | Undetermined | ND | 5.16 | ND | Undetermined | 33.89 | Undetermined | ND | 0.40 | ND |
| **19** | Undetermined | 21.25 | Undetermined | ND | >1000 | ND | Undetermined | 34.70 | Undetermined | ND | 0.23 | ND |
| **21** | Undetermined | 21.70 | Undetermined | ND | >1000 | ND | Undetermined | 35.07 | Undetermined | ND | 0.18 | ND |
| **24** | Undetermined | 29.63 | Undetermined | ND | 8.96 | ND | Undetermined | 33.13 | Undetermined | ND | 0.66 | ND |
| **26** | Undetermined | 24.98 | Undetermined | ND | 154.01 | ND | Undetermined | 33.39 | Undetermined | ND | 0.55 | ND |
| **29** | Undetermined | 23.10 | Undetermined | ND | 483.35 | ND | Undetermined | 33.37 | Undetermined | ND | 0.56 | ND |
| ND: Not detected; n/a: not available | | | | | | | | | | | | |
